# Special Characterization and Excellent Antioxidant Capabilities of Zinc Chelated Squid Protein Nanoparticles

**DOI:** 10.3390/foods14101789

**Published:** 2025-05-18

**Authors:** Qiyi Zhou, Tianming Wang, Lixin Liu, Yaqi Kong, Yifan Liu, Wenhui Wu, Xiaozhen Diao

**Affiliations:** 1 Department of Marine Biopharmacology, College of Food Science and Technology, Shanghai Ocean University, Shanghai 201306, China; zhouqiyi_971@yeah.net (Q.Z.); wtm4411@163.com (T.W.); 18651125617@163.com (L.L.); m230351113@st.shou.edu.cn (Y.K.); tutti77@163.com (Y.L.); 2 Putuo Sub-Center of International Joint Research Center for Marine Biological Sciences, Zhongke Road, Putuo District, Zhoushan 316104, China

**Keywords:** squid protein peptides, preparation, peptide-zinc chelates, antioxidant evaluation, structural characterization

## Abstract

The functional exploration of marine-derived proteins is at the forefront of nutritional research. The Argentine squid protein (ASP) was extracted from Argentine squid carcasses and was hydrolyzed using neutral protease, with the degree of hydrolysis serving as the response variable. Using single-factor experiments and response surface methodology, we identified optimal conditions for preparing Argentine squid protein peptides (ASPP). The hydrolysis degree reached 41.32% ± 0.27 under the conditions of 7% enzyme preparation addition, 2.4 h enzyme digestion time, and 6% substrate concentration. The ASPP was subsequently chelated with zinc sulfate to produce Zn-ASPP, whose structural and functional properties—including particle size, FTIR, DSC, viscosity, SEM, solubility, emulsibility, foamability, and antioxidant capacity—were systematically characterized. The results indicate that Zn-ASPP forms stable nanoparticles with strong antioxidant activity. The strongest antioxidant capacity reached 73.79% at a solution pH of 8, making it particularly valuable for food industry applications. This work may provide a theoretical basis and practical guidance for the development of zinc-fortified marine protein supplements with enhanced antioxidant properties.

## 1. Introduction

*Illex argentinus*, also known as gun squid, is notable for its high protein content and balanced amino acid composition, classifying it as a low-fat, high-protein seafood with substantial nutritive and developmental potential [1]. Previous reports indicate that squid protein accounts for approximately 16%–18% of its total mass and is rich in the eight essential amino acids required by the human body [2]. The composition of essential amino acids is close to that of the total protein, making it more readily digested and absorbed than many conventional animal proteins and many other aquatic proteins (such as fish meal and crab protein) [3]. Moreover, the hydrolysis of squid muscle can yield five unique amino acids with antioxidant properties [4]. In addition, compared with common aquatic proteins such as salmon, squid protein is rich in trace elements (such as selenium and zinc), offering higher nutritional value [5]. In terms of safety, squid is an aquatic protein that is least affected by mercury contamination among seafood products, with mercury levels far below those of some large fish (such as sharks and tuna) [6,7]. Therefore, squid can be considered an ideal choice as a high-quality protein source.

The squid carcass, which is the principal component processed by the food industry, serves as its primary site of nutrient accumulation [8]. This tissue is rich in proteins, amino acids, and taurine [9], which can exert beneficial effects such as antioxidation, cholesterol reduction, blood glucose regulation, and renal and neuronal protection [10]. Studies further suggest that taurine isolated from squid carcasses demonstrates promise in alleviating depression [11], mitigating reproductive toxicity [12], treating congestive heart failure [13], and reducing intestinal inflammation [14]. Although these findings underscore the multifaceted health benefits of squid and its byproducts, research on its deeper functional and bioactive properties remains limited.

Recent advancements in biotechnology have driven interest in squid protein peptides due to their unique biological activities. For instance, previous studies have hydrolyzed collagen and myofibrillar proteins from Argentine squid skin using three distinct proteolytic enzymes, yielding peptides with angiotensin I–converting enzyme (ACE) inhibitory, dipeptidyl peptidase-IV (DPP-IV) inhibitory, and prolyl endopeptidase (PEP) inhibitory activities [15].

Meanwhile, chelating metal ions with active peptides from marine organisms has emerged as a promising strategy to enhance specific biological functions [16,17]. However, research on zinc-ion-chelated squid protein peptides remains sparse, despite the well-established nutritional and physiological roles of essential minerals in regulating enzyme reactions and various metabolic processes [18]. Dietary habits, lifestyle patterns, and other factors can lead to widespread deficiencies in these mineral elements, which, in turn, may contribute to significant health concerns [19,20,21,22]. Zinc deficiency, in particular, has been linked to many health risks [23]. Conventional inorganic zinc supplements suffer from low bioavailability and gastrointestinal irritation, while those chelated with proteolytic peptides mitigate the drawbacks well, thus attracting considerable attention.

In light of these considerations, the present study aims to extract squid protein via alkali extraction and acid precipitation and optimize the hydrolysis process of squid protein peptide using a Box–Behnken response surface design. A zinc chelate peptide is subsequently formed by chelating zinc sulfate with the prepared squid protein peptide. By comparing multiple parameters—such as structural changes, application characteristics, and antioxidant capacity—among the resulting products, this work explores the potential of zinc-chelated squid protein peptides in food processing and pharmaceutical applications.

## 2. Materials and Methods

### 2.1. Materials and Reagents

Argentine squids were purchased from Zhejiang Haixin Foods. All proteases, including Trypsin (Cat. No. T8150), Chymotrypsin (Cat. No. C8660-1), and Papain (Cat. No. G8430), were purchased from Solarbio (Beijing Solarbio Science & Technology, Beijing, China). Sodium dodecyl sulfate (SDS) and Coomassie brilliant blue G250 were purchased from Macklin (Macklin Biochemical, Shanghai, China; Cat. No. C8420). The SDS-PAGE gel preparation kit was purchased from Epizyme (Epizyme Biomedical Technology, Shanghai, China; Cat. No. PG112). The Total Antioxidant Capacity (T-AOC) Assay Kit was purchased from Leagene (Leagene Biotechnology, Beijing, China; Cat. No. ST1420). The BCA Protein Assay Kit was purchased from Labgic (Labgic Technology, Beijing, China; Cat. No. BL521A). All other chemicals and reagents were commercially available and of analytical grade.

### 2.2. Preparation of Argentine Squid Protein (ASP)

As shown in Appendix A, only the carcass of the Argentine squid was retained after treatment, cut up, divided into 3 parts, then soaked for 1 d in 6× the volume of NaOH solution (0.1 M) to remove pigment and fat, and then it was washed and pureed.

Part 1: Alkaline extraction and acid precipitation (AEAP): AEAP was performed, where the protein was extracted at 4 °C using a solution volume six times that of the sample in NaOH (0.1 M) for 7 h. After centrifugation at 6000× *g* for 20 min, the supernatant was collected, and the pH was adjusted to 5.5 with HCl (6 M). A second centrifugation step was performed under the same conditions for 20 min to harvest the resulting sediment.

Part 2: Acid extraction (AE): Acid extraction was conducted by soaking the sample in an acetic acid solution (0.1 M) at 4 °C for 24 h, with a volume six times that of the sample. After centrifugation at 6000× *g* for 20 min, the supernatant was collected, and the precipitate was extracted twice. The supernatant was combined and then concentrated by rotary evaporation.

Part 3: Water extraction (WE): The squid puree was soaked in a solution containing sodium chloride (NaCl, 0.1 M) and phosphate buffer (0.02 M, pH 7.4) at 4 °C for 24 h, utilizing a volume three times that of the sample. Upon completion of the soaking, the mixture was centrifuged at 6000× *g* for 20 min to separate the supernatant from the sediment. The precipitate was extracted twice more, and the combined supernatants were subsequently adjusted to a pH of 5.5 with a 1 M HCl solution. A final centrifugation step at 6000× *g* for 20 min was employed to collect any remaining sediment.

The three protein fractions obtained from the extraction processes were subsequently dehydrated in a vacuum freeze dryer. The dried protein extracts were stored in a constant temperature drying oven for future analyses.

#### 2.2.1. Amino Acid Composition

Amino acid composition and quantification of ASP were conducted using a high-performance liquid chromatograph (HPLC) (U3000, Thermo Fisher Scientific, Waltham, MA, USA) in accordance with the methodology outlined in our previous study [24]. A total of 16 amino acids were analyzed to determine their individual concentrations and overall composition within the sample.

#### 2.2.2. Protein Extraction Rate (PER) and Protein Purity (PP)

PP was determined using the Kjeldahl apparatus (Kjeltec 8400, Foss Analytical, Denmark) as previously described with minor methodology modifications [25].

As shown in Equation (1), PER is expressed as follows:

(1) $$\mathrm{PER}(\%)=\frac{a}{b}\;\times \;100\%$$

As shown in Equation (2), PP is expressed as follows:

(2) $$\mathrm{PP}(\%)=\frac{a}{c}\;\times \;100\%$$

Note: *a* is actual protein content in ASP, *b* is mass of Argentine squid carcasses, and *c* is mass of freeze-dried ASP.

### 2.3. Preparation of Argentine Squid Protein Peptides (ASPP)

#### 2.3.1. Selection of Preparation Conditions for ASPP

A certain concentration of ASP solution was configured, and the pH and the water bath temperature were adjusted after adding a certain amount of protease preparation. The protease was heat inactivated under the conditions of 100 °C and 10 min after the hydrolysis step. Then, the degree of hydrolysis (DH) was measured after cooling.

This study was conducted by examining the effects of the following four variables: the type of protease (A), the amount of enzyme added (B), the duration of enzymolysis (C), and the concentration of the substrate (D). The response value used to assess these factors was the degree of hydrolysis (DH) (Y).

For variable (A), different types of proteases were evaluated, including pepsin, acid protease, papain, neutral protease, and trypsin. The optimal reaction temperatures and pH values for each protease are summarized in Table 1.

When assessing variable (B), the concentration gradients of the protease were set at 4%, 5%, 6%, 7%, and 8%. For variable (C), the hydrolysis times were varied to 1.2, 1.6, 2, 2.4, 2.8, and 3.2 h. Lastly, for variable (D), the concentration gradients of the protein solution were also set at 4%, 5%, 6%, 7%, and 8%.

#### 2.3.2. Response Surface Methodology (RSM)

On the basis of Section 2.3.1., a three-factor three-level Box-Behnken design (BBD) was used to optimize the preparation conditions of ASPP, and the level of factors is shown in (Table 2).

#### 2.3.3. Degree of Hydrolysis (DH)

DH was determined by the formaldehyde titration method described before [31].

As shown in Equation (3), ANC is expressed as follows:

(3) $$\mathrm{ANC}\;(\mathrm{mg}/100\;\mathrm{mL})=\frac{\left(V_{2}-V_{1}\right)\times C\times 0.014\times 100}{20\times 5}\;\times \;100$$

As shown in Equation (3), DH is expressed as follows:

(4) $$\mathrm{DH}\;(\%)=\frac{m-n}{p}\;\times \;100$$

Note: ANC is amino nitrogen content (mg/100 mL), C is concentration of standard solution of NaOH (mol/L), V_1_ is the volume of NaOH consumed by the blank group titration, V_2_ is the volume of NaOH consumed by the titrating sample, m is ANC of enzymatic hydrolysis products, n is ANC of substrate, and p is total protein nitrogen content in the substrate.

### 2.4. Preparation of Zinc-Chelated Argentine Squid Protein (Zn-ASPP)

Peptide–zinc chelates were prepared as previously reported [32].

To prepare the peptide–zinc chelate, an ASPP solution (5 mg/mL) was combined with zinc sulfate (2 mMol/L), with a volume ratio of 3:1, and stirred continuously at a temperature of 30 °C for a duration of 30 min to facilitate complex formation. Following this reaction, the resulting peptide–zinc chelates were subjected to dialysis, utilizing a semipermeable membrane with a molecular weight cut-off of 500 Da. This process, lasting for 5 h, effectively removed any unbound or free zinc ions from the solution.

After dialysis, the solution was frozen at −80 °C to preserve its structural integrity and facilitate subsequent processing. The frozen solution was then subjected to vacuum freeze-drying, which allowed for the extraction of moisture while maintaining the stability of the peptide–zinc chelate, eventually yielding the desired powder form of peptide–zinc chelate. This powder was collected for subsequent experiments.

#### Measurement and Calculation of Zinc Content and Zinc Chelation Rate

EDTA titration was used to determine the zinc content as previously reported [33].

As shown in Equation (5), zinc content is expressed as follows:

(5) $$\mathrm{Zinc}\;\mathrm{Content}\;(\mathrm{mg}/g)=\frac{M\times c\times V\times 5}{m}\;\times \;100\%$$

Note: M is the relative atomic mass of zinc, c is the concentration of EDTA standard titrant (mol/L), and m is the weighed mass of Zn-ASPP (g).

According to the method of Wang [34], the zinc chelation rate was measured by EDTA complexometric titration.

As shown in Equation (6), the zinc chelation rate is expressed as follows:

(6) $$\mathrm{Zinc}\;\mathrm{chelation}\;\mathrm{rate}\;(\%)=\frac{(V_{2}-V_{0})C}{(V_{1}-V_{0})C}\;\times \;100\%$$

Note: C is the concentration of EDTA (mol/L), V_1_ is the volume of EDTA consumed in the determination of total zinc content (mL), V_2_ is the volume of EDTA consumed in the determination of chelated zinc content (mL), and V_0_ is the EDTA volume required to replace the liquid under the test with deionized water (mL).

### 2.5. Structural Characterization

#### 2.5.1. SDS-PAGE

The electrophoresis gel was prepared using a 12.5% SDS-PAGE gel rapid preparation kit (Cat. No. PG213, Epizyme Biomedical Technology, Shanghai, China). The sample was dissolved in Milli-Q water, and 4 × sample loading buffer was mixed thoroughly into the sample solution. The sample was heated in a metal bath at 100 °C for 5 min and then briefly centrifuged.

We loaded 10 μL of the sample and 5 μL of the marker (10–250 kDa) (Cat. No. WJ103L, Epizyme Biomedical Technology, Shanghai, China) into the gel well. Initially, the voltage was set to 80 V, and it was increased to 120 V once the sample separated from the gel. After electrophoresis, the gel was stained with Coomassie Brilliant Blue (Cat. No. C8430, Solarbio, Beijing, China) for 30 min. Finally, the eluent (Cat. No. SL1300, Epizyme Biomedical Technology, Shanghai, China; Ethanol:Glacial Acetic Acid:ddH_2_O = 250 mL:80 mL:670 mL) was used until the electrophoretic bands became clear. Eventually, the gel was imaged with the Amersham Imager 600 System (Cytiva, Marlborough, MA, USA).

#### 2.5.2. Scanning Electron Microscope (SEM)

The microstructures of ASP, ASPP, and Zn-ASPP chelates were examined using a scanning electron microscope (Zeiss Gemini Sigma 300 VP SEM, Carl Zeiss AG, Jena, Thuringia, Germany). Prior to imaging, the samples were placed on a glass slide using double-sided tape. To enhance electrical conductivity, gold and platinum were sprayed onto the samples. The imaging magnifications were set at 500×, 2K, and 5K.

#### 2.5.3. Differential Scanning Calorimetry (DSC)

The thermal stabilities of ASP, ASPP, and Zn-ASPP were investigated with the aid of a differential scanning calorimeter (DSC) (Netzsch DSC 200 F3, Carl Zeiss AG, Thuringia, Germany). The temperature range was set at 30~200 °C, the heating rate was 5 °C/min, and the heating began in a nitrogen atmosphere (purity ≥ 99.9%).

#### 2.5.4. Fourier Transform Infrared Spectroscopy (FTIR)

A Fourier transform infrared spectrometer (FTIR) (Nicolet 6700, Wisconsin, America) was used to acquire spectra to characterize the secondary structures of ASP, ASPP, and Zn-ASPP. With the initial air as the background, the infrared spectral signal of the sample was accumulated by 32 scans using the attenuated total reflection (ATR) attachment, the test frequency range was 4000 cm^−1^~400 cm^−1^, and the resolution was 2 cm^−1^. Finally, OMNIC software (OMNIC 9, Thermo Fisher Technology, Waltham, MA, USA) was used to process the data.

### 2.6. Evaluation of Physical Property

#### 2.6.1. Particle Size (PS)

The particle sizes of ASPP and Zn-ASPP were determined using a nano particle size and zeta potential analyzer (Malvern Zetasizer Nano ZS90, Malvern Panalytical, Malvern, UK). The particle size of ASP was determined using a laser particle sizer (Malvern Mastersizer 3000, Malvern Panalytical, Malvern, UK). The samples were dissolved and ultrasonicated for 5 min, and the supernatant was centrifuged at high speed in a cuvette to determine the distribution of particle diameter size in the solution.

#### 2.6.2. Viscosity

Referring to the method of Wei [35], a rheometer (MCR 302, Anton Paar, Graz, Austria) was used to determine the shear rate profile of ASP, ASPP, and Zn-ASPP. Then, 25 mm parallel plates were selected, and the shear rates ranged from 0.001 to 1000 s^−1^ and were tested at room temperature.

### 2.7. Evaluation of Functional Properties

#### 2.7.1. Solubility

The method of Petrucelli [36] was referred to with slight modification to determine the solubility. Briefly, the pH of the sample solution was adjusted to 2, 4, 6, 8, 10, and 12, respectively, and the supernatant was taken after centrifugation to determine the protein content using the BCA kit method (Cat. No. BL521A, Biosharp, Beijing, China) to calculate the solubility.

#### 2.7.2. Antioxidant Capacity of Zn-ASPP

A T-AOC assay kit was used to determine the antioxidant capacity of Zn-ASPP. Briefly, the ABTS solution and Trolox standard gradient solution were first configured. Then the sample solution took pH (factor A) and concentration (factor B) as single variables. When (factor A) was a variable, pH was set as 2, 4, 6, 8, 10, and 12. When (factor B) was a variable, the concentration was set to 0.1, 0.2, 0.3, 0.4, 0.5, 0.6, 0.7, 0.8, 0.9, 1.0, 1.1, 1.2, and 1.4 mg/mL. After the sample was added into the prepared working fluid and reacted at 30 °C for 5 min, the absorbance value (A_405_) was measured with an microplate meter, and the standard curve was drawn.

#### 2.7.3. Foamability and Foam Stability

Foaming properties and foam stability were determined according to the methods described before [37]. Briefly, the sample stock solution was homogenized at 8000 rpm for 1 min, and the total volumes of solution and foam were measured at 0 min and 30 min of standing time, respectively. Foaming capacity (%) and foam stability (%) were calculated.

#### 2.7.4. Emulsibility and Emulsion Stability

The method of Pearce [38] was referred to and slightly modified for the determination of emulsibility and emulsion stability by the turbidimetric method. Briefly, the protein solution was mixed with soybean oil and homogenized at 8000 rpm for 1 min, and the samples were mixed with SDS (0.1%) at 0 and 10 min, respectively, and the absorbance values of the samples were measured at 500 nm, using SDS as a blank control to calculate the emulsification activity index (EAI) and emulsification stability index (ESI).

### 2.8. Statistical Analysis

The experiments were presented as means (standard deviations, SDs) in triplicate, and all data were analyzed by SPSS (version 17.0, IBM Corporation, Armonk, NY, USA). For comparing significance between multiple groups, analysis of variance (ANOVA) followed by a Tukey post-test was performed. The differences were considered significant at *p* < 0.05. Design Expert (Version 11.0, Stat-Ease, Minneapolis, MN, USA) was used for RSM, and Origin (Version 2019, OriginLab Corporation, Northampton, MA, USA) was used for mapping.

## 3. Results

### 3.1. Amino Acid Composition of ASP

According to the results presented in Table 3, the essential amino acids in ASP were notably high in lysine (7.07 g/100 g) and leucine (6.99 g/100 g). The proportion of essential amino acids in relation to the total amino acids was a significant 43.64%, aligning with the ideal structural model for high-quality proteins as outlined by the World Health Organization (WHO) [39]. Furthermore, the ratio of essential amino acids to non-essential amino acids is 77.45%, which exceeds the standards set by the FAO/WHO amino acid model [40]. When compared to salmon, both sources exhibit similar amino acid structures and appropriate ratios of essential to non-essential amino acids, providing a well-balanced nutritional profile [41]. Thus, it can be concluded that the ASP extracted from Argentine squid is a high-quality protein source.

### 3.2. Protein Extraction Rate (PER) and Protein Purity (PP)

**Figure 1:**
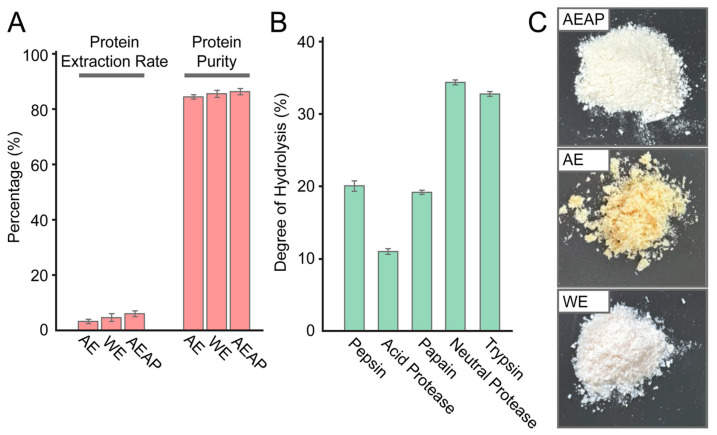
Comparison and apparent display of squid protein extraction methods. (**A**) is the protein extraction rate and purity of ASP prepared by different methods. AE: alkaline extraction and acid precipitation, WE: water extraction, AEAP: alkaline extraction and acid precipitation. (**B**) is the appearance of squid protein prepared by different methods. The protein with fine silty and slight yellowing was prepared by the AEAP method. The protein with golden color and loose texture was prepared by the AE method. The fine silty, slightly red, protein was prepared for WE. (**C**) is the hydrolysis effect of ASP under different proteases.

According to the test results of protein extraction rates (PER) and protein purity (PP) shown in Figure 1A, we found that the AEAP method yielded the best extraction results and the highest protein purity. However, the test results for ASP obtained from the three methods did not show significant differences. The overall protein extraction rate was relatively low, primarily due to the high water content found in squid, which we measured to be approximately 80.58%.

The muscle tissue of squid is dense, and the abundance of interstitial cells makes it difficult to completely break down the muscle tissue, contributing to the low protein extraction rate. Despite this, the protein purity levels were generally high, exceeding 84%. Notably, the ASP results obtained using the AEAP method reached a purity level of 86.32%. This indicates that the protein produced by this method has low impurity content and high nutritional value, making it suitable for the development of high-end protein raw materials. The protein powder produced by the three methods is illustrated in Figure 1B.

### 3.3. Preliminary Hydrolysis Condition

According to the DH of ASP under different proteases (Figure 1B), it can be seen that, under the conditions of a substrate concentration of 5%, an enzymolysis time of 4 h, and an enzyme preparation addition amount of 9%, neutral protease is the most suitable protease for preparing ASPP, since it possesses the highest hydrolysis degree.

**Figure 2:**
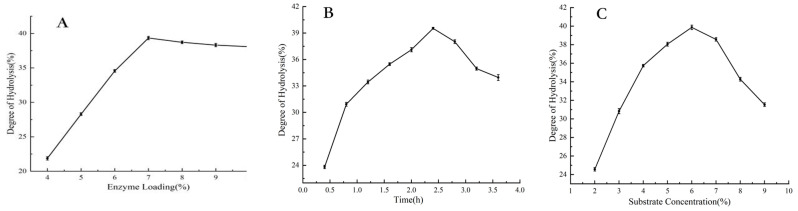
Test results of the main factors influencing hydrolysis degree: (**A**) the effects of enzyme addition, (**B**) hydrolysis time, (**C**) substrate concentration on the hydrolysis of ASP.

Figure 2A illustrates the effect of neutral protease dosage on the degree of hydrolysis (DH). It was determined that a 7% enzyme preparation dosage was optimal, resulting in the highest DH. When the enzyme preparation dosage was increased beyond this level, the DH did not change significantly. This lack of change may be attributed to the substrate being fully consumed at higher enzyme concentrations, which can lead to excessive enzyme self-hydrolysis, reducing the DH.

The impact of enzymatic hydrolysis time on the DH is presented in Figure 2B. The results indicate that 2.4 h is the ideal duration for hydrolysis, as the protease is exhausted at this point, yielding the highest DH. As the hydrolysis time increases further, the peptides produced during the process begin to act as substrates in the enzymatic reaction, decreasing the efficiency of hydrolysis.

Regarding the influence of substrate concentration on the hydrolysis effect of ASP (illustrated in Figure 2C), it was observed that the DH initially increased with the rising substrate concentration but then began to decline. This decline can be explained by the fact that, as the substrate concentration increases, the binding sites between the enzyme and substrate become saturated. Additionally, the viscosity of the system increases, hindering optimal contact between the enzyme and substrate. Therefore, a substrate concentration of 6% was selected for optimal results.

### 3.4. Optimal Hydrolysis Conditions Under RSM

Based on Section 3.3, Design Expert was utilized to create a Box–Behnken design with three factors and three levels (see Table 4). The results are presented in Table 5. A multinomial fitting regression was conducted on the data obtained, leading to the derivation of a quadratic multinomial regression model equation.

We obtained the following equation:

Y = 41.59 + 0.635A − 0.0863B − 0.0613C + 0.7175AB + 0.1325AC + 0.485BC − 2.149A^2^ − 2.1B^2^ − 0.8870C^2^.

The linear regression model was found to be highly significant (*p* < 0.01), while the mis-stated item showed no significance. The regression coefficients were R^2^ = 0.9875 and adjusted R^2^_Adj_ = 0.9714, both of which exceeded 0.8799. This indicates that the model is well-fitted and highly significant. The effects of factors AB, BC, A^2^, B^2^, and C^2^ were also found to be significant. The binding F value correlates with the influence of these factors on the response variable, illustrating that the degree of influence of each factor on hydrolysis is ranked as follows: A > B > C.

**Figure 3:**
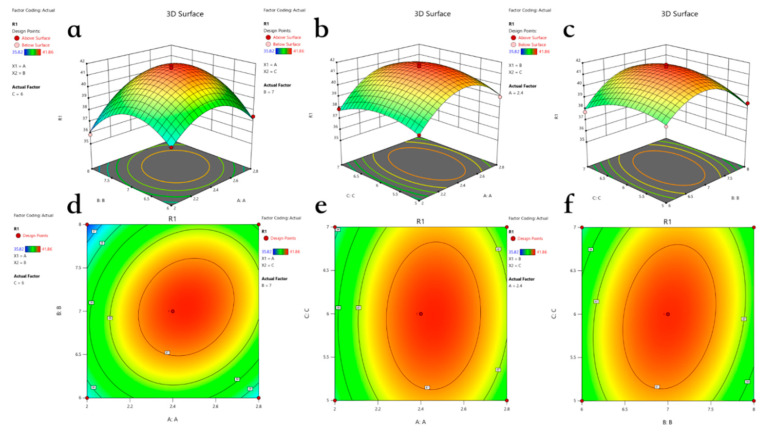
The three-dimensional response surface curves and contours show the interaction of various factors on DH. The three factors are enzyme dosage (A), enzymolysis time (B), and substrate concentration (C). (**a**–**c**) The three-dimensional response surface curves of the interaction effects of AB, AC, and BC on DH. (**d**–**f**) Contour maps of the interaction effects of AB, AC, and BC on DH.

The interaction response surface and contours of the pairwise factors are illustrated in Figure 3. The model reveals a typical convex surface characterized by inward contraction and an upward numerical trend. We observed that, as the values of the three factors increased, the degree of hydrolysis initially rose and then declined. The optimal conditions for enzymolysis were identified as follows: an enzyme dosage of 7.047, an enzymolysis time of 2.423 h, and a substrate concentration of 5.941%. However, to simplify the process, these were adjusted to 7%, 2.4 h, and 6%, respectively. Notably, the model predicted a hydrolysis value of 41.646%, which closely aligns with the actual value of 41.32%, confirming the model’s reliability.

### 3.5. Zinc Content and Zinc Chelation Rate

The zinc content in Zn-ASPP was measured at 7.60 mg/g, with a zinc chelation rate of 29.66%. There has been considerable research on peptides and their ability to chelate metals. For instance, Wei reported that the chelation rate of SDF (soluble dietary fiber) and zinc sulfate was 25.18%, with a zinc content of 465.2 µg/g achieved at 65 °C and a pH of 8.0 over a period of 120 min, using a mass ratio of 1:1 [42]. In comparison, Zn-ASPP demonstrated a significantly higher zinc content and chelation rates in a shorter reaction time under milder conditions, indicating that it is a more effective zinc-chelating agent than SDF.

Additionally, the relatively low zinc content and chelation rate of Zn-ASPP enhance its safety, making it more suitable for long-term use and easier to control in terms of dosage. High zinc levels can lead to excessive intake, which may cause health issues such as anemia, lipid metabolism disorders, and gastrointestinal reactions [43]. Therefore, Zn-ASPP offers clear advantages in terms of production efficiency and safety, achieving an effective chelation in a short time while minimizing the potential risks associated with excessive zinc intake.

### 3.6. Structural Characterization

#### 3.6.1. SDS-PAGE

Previous studies have shown that the most important protein in squid muscle is myofibrillar protein, which accounts for 60% to 70% of the total protein, including Myosin heavy chain (MHC), Actin (AC), Tromyosin (TM), and Paramyosin (PM) [44].

**Figure 4:**
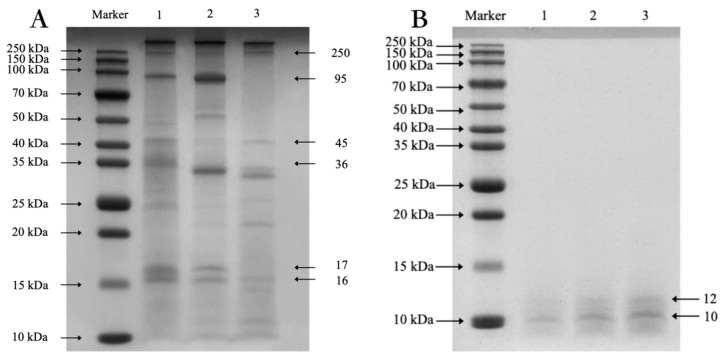
SDS-PAGE electropherogram of ASP (**A**) prepared by different methods. Lane 1: AEAP method, lane 2: AE method, lane 3: WE method. SDS-PAGE electropherograms of different concentrations of ASPP (**B**).

According to the SDS-PAGE results (Figure 4A), it can be seen that there are six main bands of ASP, which are Myosin Heavy Chain (MHC) with a molecular weight of 250 kDa, Paramyosin (PM) with a weight of about 95 kDa, Actin (A) with a weight of about 45 kDa, Tromyosin (TM) with a weight of about 36 kDa, Myosin Light Chains (MLC) with a weight of about 17 kDa [45], and Troponin T (TNT) with a weight of about 16 kDa [46], and the rest are regulatory proteins [47], which is consistent with the results reported before [48,49].

In lane 2, the color of the band AC is relatively lighter, indicating that the AC is partially degraded under acidic conditions. The advance of the TM bands suggests that protein degradation is taking place. In lane 3, we observed the absence of PM bands. This could be attributed to PM being a fibrous protein with strong hydrophobicity, which may not be effectively extracted using the water extraction method. In contrast, the strip in lane 1 is clear, with no aggregation or degradation of macromolecular proteins due to acid-based treatment. This indicates that the modified preparation method is the most effective.

Using ImageJ Fiji distribution (version 2.7.0, NIH, Bethesda, MD, USA) [50], we determined that approximately 7.89% of the strips have a molecular weight above 150 kDa, while 92.11% have a molecular weight below that threshold.

**Figure 5:**
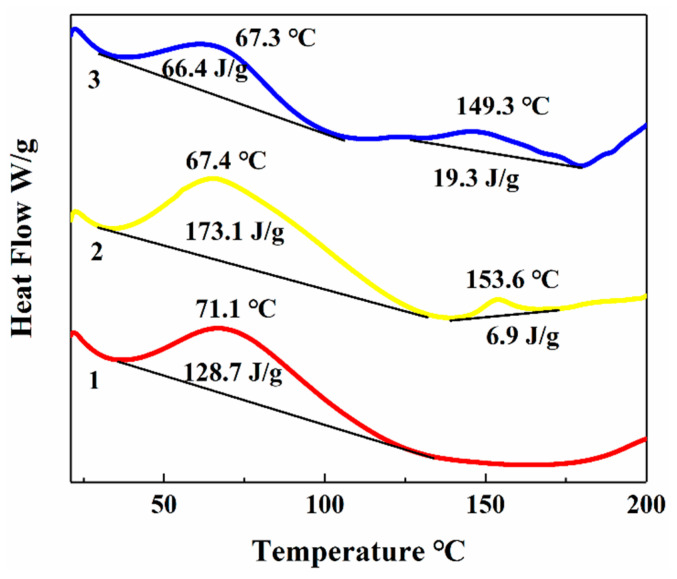
Thermogravimetric profiles of ASP, ASPP, and Zn-ASPP. Line 1: ASP, line 2: ASPP, and line 3 stands for the thermogravimetric profile of Zn-ASPP.

Figure 4B displays the SDS-PAGE electropherograms for different concentrations of the ASPP solution, revealing that the molecular weight of ASPP is below 15 kDa, with the main bands appearing at 12 kDa and 10 kDa. Comparing this with Figure 3b and Figure 5, we conclude that ASP was successfully hydrolyzed to ASPP, and the hydrolysis effect is satisfactory.

#### 3.6.2. Microscopic Morphology of ASP, ASPP, and Zn-ASPP

**Figure 6:**
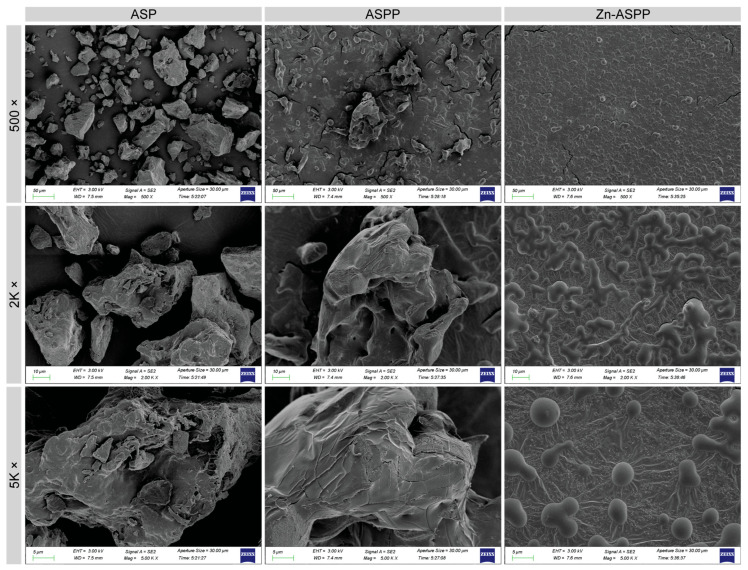
Scanning electron micrographs of ASP, ASPP, and Zn-ASPP. The images are enlarged 500, 2k, and 5k times from top to bottom, respectively.

The SEM results are presented in Figure 6, revealing distinct differences in the electron micrographs of ASP, ASPP, and Zn-ASPP. ASP features large particles with sparse distribution and a rough, blocky surface structure. In comparison, ASPP consists of smaller particles that exhibit a laminar or blocky structure, along with sparse texture, surface cracks, and fewer pores. This may result from water loss during the vacuum freeze-drying process.

On the other hand, Zn-ASPP displays the smallest particles, a dense distribution, a smooth surface, and a structure that is either spherical or interwoven. This compact globular structure may be attributed to chelation, which causes the peptide chains to fold tightly. During the drying process, particularly after chelation, protein aggregation occurs as the interactions between protein molecules change, affecting the aggregation mode and morphology. Furthermore, the use of spray drying techniques can lead to the instantaneous drying of solution droplets, which may appear as interwoven globular structures when viewed under electron microscopy.

#### 3.6.3. Thermal Stability of ASP, ASPP, and Zn-ASPP

The thermodynamic properties of ASP, ASPP, and Zn-ASPP were studied using differential scanning calorimetry (DSC). By analyzing the DSC curves, we can identify the thermodynamic changes that occur during protein hydrolysis and chelation, which allows us to assess the success of the reaction and to evaluate the thermal stability of the samples.

The melting peak temperature of ASP (71.09 °C) is higher than that of ASPP (67.42 °C), indicating that ASP undergoes structural changes at a greater temperature, suggesting good thermal stability. Additionally, the enthalpy change (∆H) value for ASP is 128.7 J/g, which is less than that of ASPP at 173.1 J/g, further indicating good thermal stability for ASP. This comparison also suggests that hydrolysis leads to the formation of smaller peptides, as the weakening of intermolecular forces due to hydrolysis results in a lower melting point. Furthermore, the breaking and formation of chemical bonds during hydrolysis are accompanied by the absorption and release of energy, which contribute to the increase in enthalpy change.

For Zn-ASPP, the melting point (67.34 °C) is slightly lower than that of ASP and is comparable to that of ASPP. However, its enthalpy change (66.39 J/g) is significantly lower than that of the other two, indicating that Zn-ASPP has the best thermal stability. This notable decrease in enthalpy change may suggest a change in the way zinc ions interact with protein peptides during chelation, affecting energy release or absorption.

The crystallinity of Zn-ASPP (149.34 °C) is lower than that of ASPP (153.61 °C), which indicates that the chelation of zinc ions reduces the crystallization temperature of protein peptides. Moreover, the enthalpy change (19.33 J/g) is significantly higher than that of ASPP (6.912 J/g), suggesting that after zinc chelation, the crystallization process of the protein peptide becomes more pronounced, resulting in a more stable crystal structure and a higher degree of crystallization.

In conclusion, zinc chelation effectively enhances the thermal stability and crystallization properties of ASPP, making Zn-ASPP not only thermally stable but also providing a more stable crystal structure. This could allow Zn-ASPP to be used as a high-quality functional food additive or drug carrier in the fields of food and medicine, improving the stability and bioavailability of various products.

#### 3.6.4. Structural Changes of ASP, ASPP, and Zn-ASPP Based on FTIR

**Figure 7:**
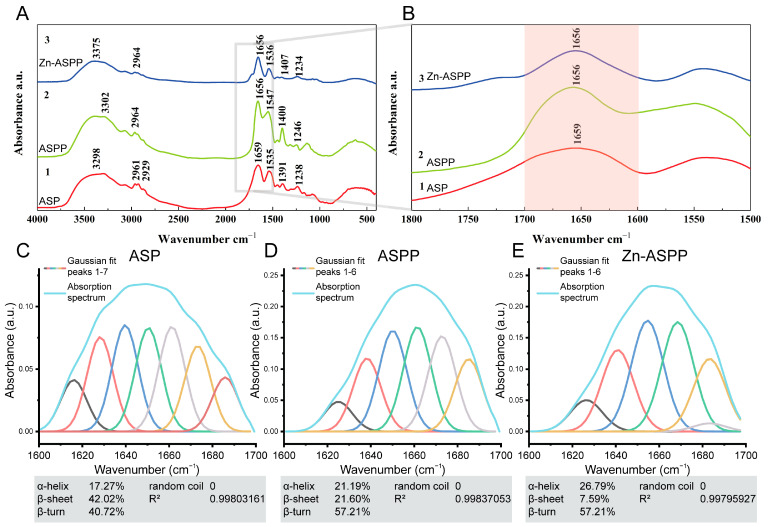
Infrared absorption spectra and Gaussian fitting diagram of ASP, ASPP, and Zn-ASPP. In the infrared absorption spectra (**A**) and local magnification (**B**), line 1 is ASP, line 2 is ASPP, and line 3 is Zn-ASPP. Gaussian fitting diagram of ASP (**C**), ASPP (**D**), Zn-ASPP (**E**).

The structural changes during the transformation of ASP, ASPP, and Zn-ASPP were identified by FTIR (Figure 7A), and the positions of the amide bands and the arrangement of the dependent groups are shown in Table 6.

The amide A band usually exhibits N-H stretching vibration in the range of 3400–3440 cm^−1^ [51]. However, the amide A bands of ASP, ASPP, and Zn-ASPP appear at 3298, 3302, and 3375 cm^−1^, respectively, which may be due to the formation of intermolecular hydrogen bonds, which leads to the redshift of the stretching vibration peak of N-H bonds [55]. In addition, the strong electron attraction effect of Zn^2+^ during chelation also causes the stretching of hydrogen bonds [56]. The typical absorption range of the amide II band is 1550–1600 cm^−1^, while the absorption peaks of the three are at 1535, 1547, and 1536 cm^−1^, respectively, all at low frequencies, indicating that the protein derived from Argentine squid has a high degree of hydrogen bonding [57]. In ASPP, 2964 cm^−1^ is the amide B band, which is caused by C-H stretching vibration [52]; 1656 cm^−1^ is the amide I band, which is caused by C=O stretching vibration [53]; 1547 cm^−1^ and 1246 cm^−1^ are the amide II band and the amide III band, which are related to N-H bending and C-N stretching vibration [54]; and the absorption peaks of the above amide bands are offset compared with ASP, indicating that the structure of ASP changes significantly after hydrolysis. After chelating zinc, the amide A band of Zn-ASPP moved back to 3375 cm^−1^ due to the high frequency absorption of N-H stretching vibration, and zinc ions formed coordination bonds with nitrogen or oxygen atoms, which changed the electronic environment of N-H groups and increased the vibration frequency, indicating that the electron cloud density of N-H bonds was weakened due to the formation of N-Zn [58]. In addition, the absorption peaks of amide II and amide III bands were also shifted after chelation, indicating that N-H and C-N bonds reacted with Zn^2+^.

It has been reported that proteins have several characteristic absorption bands in the infrared region, and the amide I band is the most valuable for studying the secondary structure [59]. Therefore, secondary structure patterns were determined using PeakFit (version 4.12, SPSS, Chicago, IL, USA) and Gaussian peak fitting algorithm. The results showed that the α-helical content of ASP (Figure 7C), ASPP (Figure 7D), and Zn-ASPP (Figure 7E) were 17.27%, 21.19%, and 26.79%, respectively. An increase in the proportion of α-helical structures is usually associated with an increase in protein stability [60]. The gradual increase in α-helix content during the transition from protein to chelate indicates that the structure of the protein becomes more stable. The β-folding content of 42.02%, 21.60%, and 7.59% showed a significant decrease in the content, indicating a change in the folding pattern of the protein. The β-turning content of 40.72%, 57.21%, and 45.94% was observed. The content increased and then decreased, and this change suggests that the structure becomes looser during the conversion of the protein to peptide and may be re-stabilized after chelation with zinc. The above results show that the structure of the samples in the three stages obviously changed, which proves the occurrence of enzymatic hydrolysis and chelation process. In addition, no random curling was detected in the structure of the three samples, indicating that the sample preparation process did not cause protein denaturation.

### 3.7. Physical Property

#### 3.7.1. Particle Size of ASP, ASPP, and Zn-ASPP

**Figure 8:**
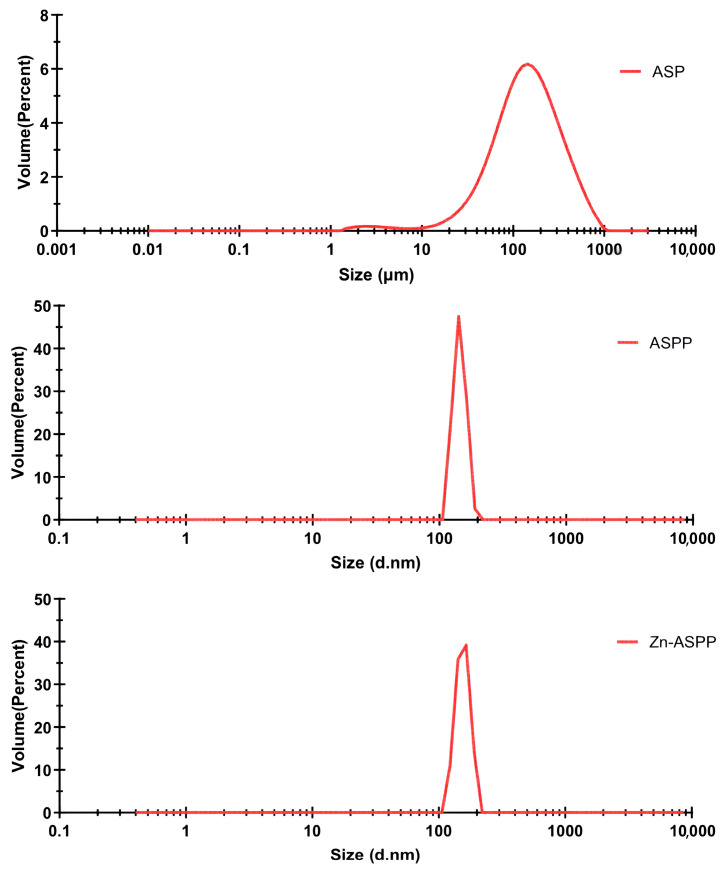
The particle size distribution of ASP, ASPP, and Zn-ASPP.

Particle size is a crucial physical parameter for characterizing metal chelates [61]. From the particle size distribution analysis presented in Figure 8, it can be observed that the average particle sizes for ASP, ASPP, and Zn-ASPP are 148.816 μm, 144.9 nm, and 155.3 nm, respectively. Notably, the average particle size of Zn-ASPP is 7.18% larger than that of ASPP, which provides evidence that ASPP effectively chelates with Zn^2+^. This interaction not only enhances the signals but also suggests the presence of both intramolecular and intermolecular chelation reactions. Furthermore, the increased particle size implies that the peptide and its corresponding chelate are distinct entities rather than homologous substances.

The Polymer Dispersity Index (PDI) serves as an indicator of the homogeneity of particle sizes within a system, where a smaller PDI value signifies a more uniform particle size distribution and a higher concentration of the particle system. This is evident in the particle size distribution graphs, where smaller PDI values correlate with narrower peaks [62]. The respective PDIs for the samples were 2.5 for ASP and 0.6 for both ASPP and Zn-ASPP. The single peak distributions observed in all cases indicate that both ASPP and Zn-ASPP exhibit uniform dispersion, reflecting the concentrated nature of these systems.

#### 3.7.2. Viscosity of ASP, ASPP, and Zn-ASPP

**Figure 9:**
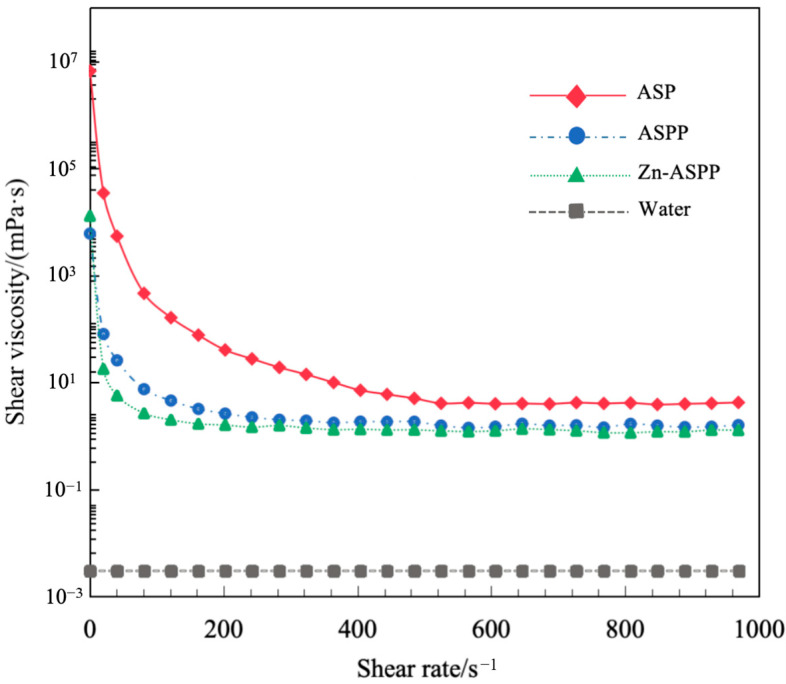
Rheological curves of ASP, ASPP, and Zn-ASPP.

The rheological behavior and intermolecular interactions of the samples can be analyzed by measuring their viscosities at different shear rates (0–1000 s^−1^). As shown in Figure 9, the viscosities of all three samples decrease rapidly with increasing shear rate and then stabilize, demonstrating strong shear-thinning characteristics. This indicates that all three samples are classified as pseudoplastic fluids [63].

At low shear rates, the initial viscosities of all three samples displayed temporarily high values due to the resistance of their spatial structures before they were deformed by force. As the shear force reached a certain level, the structures gradually collapsed, resulting in a decrease in viscosity, which can be described as shear dilution.

Among the three samples, the initial viscosity of ASP (the largest molecular structure) was the highest due to stronger intermolecular forces. In contrast, ASPP, which has a smaller molecular weight, exhibited the lowest initial viscosity, but its viscosity change was minimal, suggesting that the molecular structure of the protein peptide is more stable during the shearing process. The initial viscosity of Zn-ASPP fell between the two, likely due to the chelating effect of zinc ions that enhances intermolecular cross-linking.

### 3.8. Functional Properties

#### 3.8.1. Solubility

**Figure 10:**
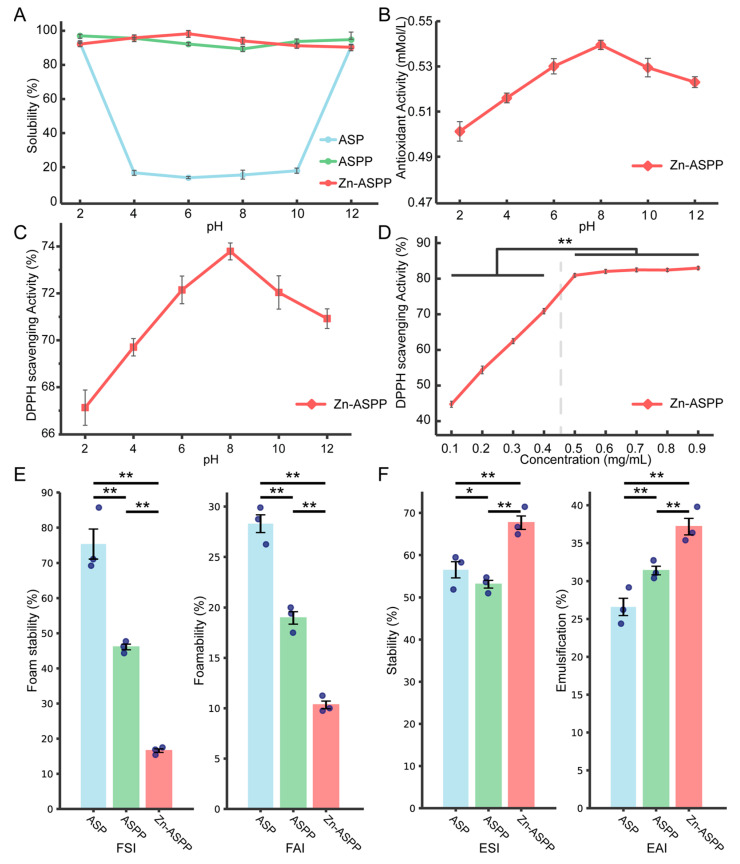
Test results of ASP, ASPP, and Zn-ASPP. The solubility of ASP, ASPP, and Zn-ASPP at different pH values (**A**). Antioxidant capacity of Zn-ASPP solutions at different pH values (**B**). The DPPH radical scavenging rate of the Zn-ASPP solution under different pH values (**C**) and different concentrations (**D**). Foamability and foam stability of ASP, ASPP, and Zn-ASPP (**E**). Emulsification and stability of ASP, ASPP, and Zn-ASPP (**F**). * *p* < 0.05, ** *p* < 0.01. (*n* = 3, one-way ANOVA and Tukey post-test).

The solubility of ASP is significantly influenced by pH levels, as shown in Figure 10A. When the pH is neutral, the solubility is very low, likely because it approaches the isoelectric point of the majority of ASP. At pH levels of 2 and 12, the solubility increases and is relatively similar; this is probably due to the dissociation of the protein molecule, which alters its secondary structure and impacts the charge of the protein through its side chain groups [64,65]. In contrast, the solubility of ASPP and Zn-ASPP is less affected by pH levels, remaining above 90%. This indicates that both compounds possess good solubility and are resilient to decomposition or precipitation under various acidic or basic conditions. The high solubility of ASPP and Zn-ASPP can also be attributed to their smaller molecular weights and simpler structures compared to ASP, which allow them to remain soluble across a broader pH range.

#### 3.8.2. Antioxidant Properties of Zn-ASPP

The antioxidant capacity of Zn-ASPP, as shown in Figure 10B, gradually strengthens with the increasing pH, peaking at a pH of 8 before gradually diminishing. This behavior may be attributed to a pH of 8 being close to the isoelectric point of Zn-ASPP, where the protein structure is stable, and the charges are neutralized, allowing for an optimal antioxidant activity.

The DPPH radical is a stable free radical that can be measured using a spectrophotometer at an absorbance of 517 nm. Changes in absorbance at this wavelength, caused by antioxidants, are commonly utilized to assess antioxidant capacity [66]. Good natural antioxidants such as vitamin C are known to have a DPPH radical scavenging rate of over 90% [67]. Synthetic antioxidants such as BHT (Butylated Hydroxytoluene) have values in the range of 60–80% [68]. Antioxidants from plant extract sources such as rosemary extract have values in the range of 60–75% [69]. In our research, it was found that the DPPH radical scavenging rate of Zn-ASPP (Figure 10C) follows a similar trend as its antioxidant capacity, remaining above 66% at all pH levels and reaching a maximum of approximately 73.79% at a pH of 8. Although lower than that of vitamin C, it was within the range of the DPPH radical scavenging rate of antioxidants from both synthetic and plant extract sources, suggesting that Zn-ASPP possesses good antioxidant properties. Its advantage lies in the fact that chelated zinc combines the function of zinc supplementation, making it suitable for the development of multifunctional nutritional supplements. In contrast, the DPPH radical scavenging rate of Zn-ASPP (Figure 10D) solutions reflects an initial increase followed by a plateau. The highest scavenging rate occurs at a concentration of 0.5 mg/mL, after which further increases in concentration do not significantly change the scavenging rate. This suggests that the radical scavenging capacity has reached a saturation point at this concentration.

Combined with the structural characterization, we infer that the unique microstructure of Zn-ASPP gives it a large specific surface area, which provides more active sites for contact with free radicals, resulting in a higher DPPH radical scavenging rate at any pH. Meanwhile, the dense particle distribution and smooth surface reduce internal defects and pores, which lowers the oxidation rate. The better thermal stability makes it less prone to structural changes or decomposition during the reaction process, which also helps to improve its antioxidant property. In addition, Zn-ASPP contains N-H bonds that provide hydrogen atoms and C=O bonds that provide electrons to react with free radicals and improve the DPPH radical scavenging rate, and the high content of β-turns makes the active site more easily exposed, further enhancing the antioxidant capacity.

Overall, these results highlight the potential of Zn-ASPP for antioxidant applications in both medicine and food production. Further research is needed to explore the detailed mechanisms and potential applications of Zn-ASPP in different fields.

#### 3.8.3. Foaming and Foam Stability

The foaming properties of the three samples, as shown in Figure 10E, were relatively similar, with ASP generating the most foam, and Zn-ASPP producing the least. However, their foam stability varied significantly. ASP maintained the most stable foam, while Zn-ASPP dissipated the foam more easily.

This difference can be attributed to the characteristics of the proteins. ASPP, as a complex macromolecular protein, exhibits better surface activity and viscosity, which contribute to improved foam formation and stability. In contrast, ASPP has a smaller molecular weight, lower viscosity, a simpler structure, weaker surface activity, and, consequently, poorer foam stability. Furthermore, the structure of the peptide in Zn-ASPP is altered due to chelation, which reduces its adsorption at the gas–liquid interface, resulting in decreased foam stability.

#### 3.8.4. Emulsification and Emulsion Stability

Emulsifying ability refers to an emulsifier’s capacity to stably disperse oil droplets when forming an emulsion in the aqueous phase [70]. As shown in Figure 10F, the emulsification effectiveness follows this order: Zn-ASPP > ASPP > ASP. Regarding emulsion stability, the order is Zn-ASPP > ASP > ASPP.

Emulsifying ability is the ability of an emulsifier to stably disperse oil droplets when forming an emulsion in the aqueous phase. Notably, Zn-ASPP demonstrated the highest emulsibility and emulsion stability, achieving 37.18% and 67.68%, respectively. This superior performance may be attributed to the enhanced surface activity of the peptide and structural alterations of the peptide chain due to chelation. Such changes improve the adsorption capacity at the oil–water interface, thereby enhancing emulsification properties [71]. Additionally, the introduction of Zn^2+^ can modify the charge distribution on the surface of ASPP, forming coordination bonds with amino acid residues. This strengthens intermolecular interactions, which helps stabilize the emulsification interface and improves emulsification stability [72].

In contrast, while ASPP initially exhibits better surface activity and emulsification, its smaller molecular weight and simpler structure may result in poorer emulsion stability over time or under varying environmental conditions. On the other hand, ASP, with its more complex three-dimensional structure, may create a thicker film at the oil–water interface, leading to weaker emulsification. However, its higher molecular weight and stronger intermolecular interactions contribute to better emulsification stability compared to ASPP.

## 4. Conclusions

In this study, we successfully extracted a squid protein peptide (ASP) from Argentine squid carcasses by screening various extraction methods. We then used Response Surface Methodology (RSM) to optimize the conditions for the enzymatic hydrolysis of ASP, resulting in high degrees of hydrolysis (DH) for ASPP. Subsequently, we prepared zinc chelate (Zn-ASPP) by combining zinc sulfate with ASPP. We conducted a series of tests to evaluate and compare the properties of all three substances.

The results indicate that Zn-ASPP not only achieves a nanometer scale but also exhibits excellent solubility, emulsification, and thermal stability. Importantly, it also demonstrates strong oxidation resistance.

This study offers a new method for preparing squid protein, squid protein peptides, and peptide zinc chelates. Additionally, it provides a scientific foundation for developing new zinc supplements aimed at specific populations. With its unique nutritional and functional attributes, Zn-ASPP shows promise as a nutritional supplement and has broad potential applications in specialized medical formulations. Future research can further investigate the role of Zn-ASPP in clinical nutrition and health foods.

## Figures and Tables

**Table 1:** Optimum reaction conditions for different proteases.

Protease	pH	Temperature/°C
Pepsin [26]	2.5	37
Acid Protease [27]	4.8	50
Papain [28]	6.0	50
Neutral Protease [29]	7.0	50
Trypsin [30]	8.0	37

**Table 2:** Factor level table.

Level	A (Enzyme Dosage/%)	B (Enzymolysis Time/h)	C (Substrate Concentration/%)
−1	6	2.0	5
0	7	2.4	6
1	8	2.8	7

**Table 3:** Amino acid composition of proteins from different sources. Despite the amino acid, EAA/NEAA: the proportion of essential amino acids to non-essential amino acids; EAA/TAA indicates the proportion of essential amino acids to total amino acids.

Amino Acid (%)	ASP (g/100 g)	Ommastrephes Bartramii (g/100 g)	Pacific Squid (g/100 g)	Salmon (g/100 g)
Aspartic acid	6.97	7.45	7.69	3.03
Threonine	3.57	3.62	3.64	1.52
Serine	3.40	3.57	3.62	1.20
Glutamic acid	8.03	8.72	9.27	4.29
Glycine	3.41	3.60	3.11	1.40
Alanine	4.69	4.86	5.04	1.94
Valine	3.42	3.35	3.25	1.62
Methionine	2.34	2.25	2.12	1.00
Isoleucine	4.00	3.97	3.94	1.49
Leucine	6.99	7.26	7.32	2.55
Tyrosine	2.84	2.80	2.60	1.14
Phenylalanine	3.96	3.89	3.40	1.48
Lysine	7.07	7.56	7.25	2.81
Histidine	1.91	2.09	1.92	0.90
Arginine	6.96	7.30	7.50	1.90
Proline	3.20	3.45	2.84	0.90
Tryptophan	0.72	0.67	0.65	0.47
Total amino acids	73.48	76.41	75.16	29.95
EAA/TAA	43.64	42.63	42	43.17
EAA/NEAA	77.45	74.29	72.42	75.97

**Table 4:** Response surface experimental design and results.

Number	A (Enzyme Dosage/%)	B (Enzymolysis Time/h)	C (Substrate Concentration/%)	Y(DH/%)
1	−1	−1	0	37.45
2	1	−1	0	37.46
3	−1	1	0	35.82
4	1	1	0	38.70
5	−1	0	−1	38.15
6	1	0	−1	38.98
7	−1	0	1	37.89
8	1	0	1	39.25
9	0	−1	−1	38.95
10	0	1	−1	38.52
11	0	−1	1	37.73
12	0	1	1	39.24
13	0	0	0	41.62
14	0	0	0	41.86
15	0	0	0	41.68
16	0	0	0	41.67
17	0	0	0	41.14

**Table 5:** Variance analysis of the regression model. Statistical analysis: one-way ANOVA and Tukey post-test *: *p* < 0.05, **: *p* < 0.01.

Variation	Square Sum	Freedom	Mean Square	F Value	*p* Value	Significance
Model	−1	−1	0	37.45		
A	1	−1	0	37.46	<0.0001 **	significant
B	−1	1	0	35.82	0.0006 **	
C	1	1	0	38.70	0.4512	
5	−1	0	−1	38.15	0.5887	
6	1	0	−1	38.98	0.0022 **	
7	−1	0	1	37.89	0.4149	
8	1	0	1	39.25	0.0157 *	
9	0	−1	−1	38.95	<0.0001 **	
10	0	1	−1	38.52	<0.0001 **	
11	0	−1	1	37.73		
12	0	1	1	39.24	0.3095	not significant
13	0	0	0	41.62		
14	0	0	0	41.86		
15	0	0	0	41.68		
16	0	0	0	41.67		
17	0	0	0	41.14		

**Table 6:** Amide band positions of ASP, ASPP, and Zn-ASPP in FTIR spectra.

Shore	Absorption Peak (cm^−1^)			Peak Assignment
	ASP	ASPP	Zn-ASPP	
Amide A	3298	3302	3375	N-H stretching [51]
Amide B	2961, 2929	2964	2964	C-H Stretch [52]
Amide I	1659	1656	1656	C=O Stretch [53]
Amide II	1535	1547	1536	N-H bending and C-N stretching [54]
Amide III	1238	1246	1234	

## Data Availability

The original contributions presented in this study are included in the article. Further inquiries can be directed to the corresponding author.

## Appendix A

The following flowchart provides detailed steps in the preparation process of ASP.

## References

[1] Dong E., Zhang X., Huang H., Chen Y., Shi S. (2020). Research progress in nutrition composition, preservation and utilization of squid. Jiangxi Fish. Sci. Technol..

[2] Shui S.-S., Yao H., Jiang Z.-D., Benjakul S., Aubourg S.P., Zhang B. (2021). The differences of muscle proteins between neon flying squid (*Ommastrephes bartramii*) and jumbo squid (*Dosidicus gigas*) mantles via physicochemical and proteomic analyses. Food Chem..

[3] Li X., Han T., Zheng S., Wu G. (2021). Nutrition and functions of amino acids in aquatic crustaceans. Adv. Exp. Med. Biol..

[4] Choi J.H., Kim K.T., Kim S.M. (2014). Optimization and biochemical characteristics of an enzymatic squid hydrolysate for manufacture of a squid complex seasoning. Food Sci. Biotechnol..

[5] Uren R.C., Bothma A. (2020). Concentrations and relative compositions of metallic elements differ between predatory squid and filter-feeding sardine from the Indian and South Atlantic oceans. Reg. Stud. Mar. Sci..

[6] Garcia Barcia L., Argiro J., Babcock E.A., Cai Y., Shea S.K.H., Chapman D.D. (2020). Mercury and arsenic in processed fins from nine of the most traded shark species in the Hong Kong and China dried seafood markets: The potential health risks of shark fin soup. Mar. Pollut. Bull..

[7] Ordiano-Flores A., Rosíles-Martínez R., Galván-Magaña F. (2012). Biomagnification of mercury and its antagonistic interaction with selenium in yellowfin tuna *Thunnus albacares* in the trophic web of Baja California Sur, Mexico. Ecotoxicol. Environ. Saf..

[8] Aubourg S.P., Trigo M., Prego R., Cobelo-García A., Medina I. (2021). Nutritional and healthy value of chemical constituents obtained from *Patagonian squid* (*Doryteuthis gahi*) by-products captured at different seasons. Foods.

[9] Matsumoto T., Akita M., Ogawa M., Goto T. (2021). Evaluation of taurine biosynthesis in the livers of the spear squid *Heterololigo bleekeri* and the swordtip squid *Uroteuthis edulis*. Fish. Sci..

[10] Fang Q., Liu J., Chen L., Chen Q., Wang Y., Li Z., Fu W., Liu Y. (2022). Taurine supplementation improves hippocampal metabolism in immature rats with intrauterine growth restriction (IUGR) through protecting neurons and reducing gliosis. Metab. Brain Dis..

[11] Zhu Y., Wang R., Fan Z., Luo D., Cai G., Li X., Han J., Zhuo L., Zhang L., Zhang H. (2023). Taurine alleviates chronic social defeat stress-induced depression by protecting cortical neurons from dendritic spine loss. Cell. Mol. Neurobiol..

[12] Owumi S.E., Popoola O., Otunla M.T., Okuu U.A., Najophe E.S. (2022). Benzo-a-pyrene-induced reproductive toxicity was abated in rats co-treated with taurine. Toxin Rev..

[13] Schaffer S., Kim H.W. (2018). Effects and mechanisms of taurine as a therapeutic agent. Biomol. Ther..

[14] Arise R.O., Adetiwa O.M., Adeoye R.I., Malomo S.O. (2022). Synergistic enhancement of rat intestinal alkaline phosphatase activity by taurine and sodium butyrate protects against endotoxin-induced bowel inflammation. J. Food Biochem..

[15] Bougatef H., Sila A., Bougatef A., Martínez-Alvarez O. (2024). Protein Hydrolysis as a way to Valorise Squid-Processing byproducts: Obtaining and identification of ACE, DPP-IV and PEP inhibitory peptides. Mar. Drugs.

[16] Hua P., Xiong Y., Yu Z., Liu B., Zhao L. (2019). Effect of *Chlorella pyrenoidosa* protein hydrolysate-calcium chelate on calcium absorption metabolism and gut microbiota composition in low-calcium diet-fed rats. Mar. Drugs.

[17] Fang Z., Xu L., Lin Y., Cai X., Wang S. (2019). The preservative potential of Octopus scraps peptides—Zinc chelate against Staphylococcus aureus: Its fabrication, antibacterial activity and action mode. Food Control.

[18] Tako E. (2022). Essential minerals: Nutritional requirements, dietary sources, and deficiencies. Nutrition Guide for Physicians and Related Healthcare Professions.

[19] Gherasim A., Arhire L.I., Niță O., Popa A.D., Graur M., Mihalache L. (2020). The relationship between lifestyle components and dietary patterns. Proc. Nutr. Soc..

[20] Guo H., Hong Z., Yi R. (2015). Core-shell collagen peptide chelated calcium/calcium alginate nanoparticles from fish scales for calcium supplementation. J. Food Sci..

[21] Guo L., Harnedy P.A., O’Keeffe M.B., Zhang L., Li B., Hou H., FitzGerald R.J. (2015). Fractionation and identification of *Alaska pollock* skin collagen-derived mineral chelating peptides. Food Chem..

[22] Guo L., Harnedy P.A., Zhang L., Li B., Zhang Z., Hou H., Zhao X., FitzGerald R.J. (2015). In vitro assessment of the multifunctional bioactive potential of Alaska pollock skin collagen following simulated gastrointestinal digestion. J. Sci. Food Agric..

[23] Ross M.M., Hernandez-Espinosa D.R., Aizenman E. (2023). Neurodevelopmental consequences of dietary zinc deficiency: A status report. Biol. Trace Elem. Res..

[24] Kong Y., Zhang L.L., Sun Y., Zhang Y.Y., Sun B.G., Chen H.T. (2017). Determination of the free amino acid, organic acid, and nucleotide in commercial vinegars. J. Food Sci..

[25] Chang S.K., Zhang Y. (2017). Protein analysis. Food Analysis.

[26] Liu X.J., Zhang Y.T., Wang X.D., Wu Y., Li K., Xu H. (2025). Effects of Different Proteases on the Enzymatic Hydrolysis and Structure of Wheat Gluten Protein. Sci. Technol. Food Ind..

[27] Wei M., Chen P., Zheng P., Tao X., Yu X., Wu D. (2023). Purification and characterization of aspartic protease from *Aspergillus niger* and its efficient hydrolysis applications in soy protein degradation. Microb. Cell Factories.

[28] Dong Y.Q., Qi J., Tian Y., Liu H., Zhang F., Liu S. (2025). Preparation and Antibacterial Activity of Antimicrobial Peptide From Grass Carp Scale. Mod. Food.

[29] Gu C.T. (2019). Preparation, Purification, and Application of Antimicrobial Peptides from Crucian Carp Scales in Fruit and Vegetable Preservation. Master’s Thesis.

[30] Perez-Velazquez M., Maldonado-Othón C.A., González-Félix M.L. (2024). Molecular Weights and Optimum Temperature and pH for Pepsin Activity of Three Sciaenid Finfish Species From the Gulf of California. Arch. Biol. Sci..

[31] Rutherfurd S.M. (2010). Methodology for determining degree of hydrolysis of proteins in hydrolysates: A review. J. AOAC Int..

[32] Udechukwu M.C., Downey B., Udenigwe C.C. (2018). Influence of structural and surface properties of whey-derived peptides on zinc-chelating capacity, and in vitro gastric stability and bioaccessibility of the zinc-peptide complexes. Food Chem..

[33] Wortmann A., Rossi F., Lelais G., Zenobi R. (2005). Determination of zinc to beta-peptide binding constants with electrospray ionization mass spectrometry. J. Mass Spectrom..

[34] Wang C., Li B., Ao J. (2012). Separation and identification of zinc-chelating peptides from sesame protein hydrolysate using IMAC-Zn^2+^ and LC–MS/MS. Food Chem..

[35] Wei Y., Cai Z., Wu M., Guo Y., Tao R., Li R., Wang P., Ma A., Zhang H. (2020). Comparative studies on the stabilization of pea protein dispersions by using various polysaccharides. Food Hydrocoll..

[36] Sanchez-Alonso I., Solas M.T., Borderías A.J. (2007). Technological implications of addition of wheat dietary fibre to giant squid (*Dosidicus gigas*) surimi gels. J. Food Eng..

[37] Wang H., Guo W., Zheng C., Wang D., Zhan H. (2017). Effect of temperature on foaming ability and foam stability of typical surfactants used for foaming agent. J. Surfactants Deterg..

[38] Pearce K.N., Kinsella J.E. (1978). Emulsifying properties of proteins: Evaluation of a turbidimetric technique. J. Agric. Food Chem..

[39] Xiccato G., Trocino A. (2010). Energy and protein metabolism and requirements. Nutrition of the Rabbit.

[40] Peres H., Oliva-Teles A. (2006). Effect of the dietary essential to non-essential amino acid ratio on growth, feed utilization and nitrogen metabolism of European sea bass (*Dicentrarchus labrax*). Aquaculture.

[41] Deng L., Li H., Jiang J. (2012). Nutrition evaluation of Norway salmon. Sci. Technol. Food Ind..

[42] Wei C., Wang X., Jiang X., Cao L. (2023). Preparation of quinoa bran dietary fiber-based zinc complex and investigation of its antioxidant capacity in vitro. Front. Nutr..

[43] Dong Y., Li D., Bao Z., Chen Y., Lin S. (2024). Mechanism of targeted uric acid reduction by soybean protein peptide SHECN in hyperuricemia mice and improvement of liver injury. Food Biosci..

[44] Raman M., Mathew S. (2014). Study of chemical properties and evaluation of collagen in mantle, epidermal connective tissue and tentacle of Indian Squid, *Loligo duvauceli* Orbigny. J. Food Sci. Technol..

[45] Highashihara M., Frado L., Craig R., Ikebe M. (1989). Inhibition of conformational change in smooth muscle myosin by a monoclonal antibody against the 17-kDa light chain. J. Biol. Chem..

[46] Vylegzhanina A.V., Kogan A.E., Katrukha I.A., Koshkina E.V., Bereznikova A.V., Filatov V.L., Bloshchitsyna M.N., Bogomolova A.P., Katrukha A.G. (2019). Full-size and partially truncated cardiac troponin complexes in the blood of patients with acute myocardial infarction. Clin. Chem..

[47] Blanco-Pascual N., Fernández-Martín F., Montero P. (2014). Jumbo squid (*Dosidicus gigas*) myofibrillar protein concentrate for edible packaging films and storage stability. LWT-Food Sci. Technol..

[48] Chen T.-T., Cui Z.-H., Bao H.-R., Guo Q.-Y. (2023). Study on thermal processing characteristics of two kinds of squid carcass meat. Food Ferment. Ind..

[49] Niu Y., Chen J., Fan Y., Kou T. (2021). Effect of flavonoids from *Lycium barbarum* leaves on the oxidation of myofibrillar proteins in minced mutton during chilled storage. J. Food Sci..

[50] Abràmoff M.D., Magalhães P.J., Ram S.J. (2004). Image processing with ImageJ. Biophotonics Int..

[51] Kang H., Zou L., Zhang H., Cai C., Wang B., Ke H. (2018). Effect of high temperature treatment on chemical forces of beef proteins and structure of myofibrillar protein. Food Sci..

[52] Tang C.-Q., Lin K., Zhou X.-G., Liu S.-L. (2016). In situ detection of Amide A Bands of proteins in water by Raman Ratio Spectrum. Chin. J. Chem. Phys..

[53] Jin Y., Su Z. (2011). Water diffusion in polycarbonate film studied by 2D FTIR spectroscopy. Chin. J. Appl. Chem..

[54] Singh B.R., DeOliveira D.B., Fu F.-N., Fuller M.P. Fourier transform infrared analysis of amide III bands of proteins for the secondary structure estimation. Proceedings of the Biomolecular Spectroscopy III.

[55] Doyle B.B., Bendit E., Blout E.R. (1975). Infrared spectroscopy of collagen and collagen-like polypeptides. Biopolym. Orig. Res. Biomol..

[56] Maiti K.S. (2018). Ultrafast N–H vibrational dynamics of hydrogen-bonded cyclic amide reveal by 2DIR spectroscopy. Chem. Phys..

[57] Nazeer A.A., Al Sagheer F., Bumajdad A. (2020). Aramid-zirconia nanocomposite coating with excellent corrosion protection of stainless steel in saline media. Front. Chem..

[58] Wang X., Zhou J., Tong P., Mao X. (2011). Zinc-binding capacity of yak casein hydrolysate and the zinc-releasing characteristics of casein hydrolysate-zinc complexes. J. Dairy Sci..

[59] De Meutter J., Goormaghtigh E. (2021). Amino acid side chain contribution to protein FTIR spectra: Impact on secondary structure evaluation. Eur. Biophys. J..

[60] Modi T., Campitelli P., Kazan I.C., Ozkan S.B. (2021). Protein folding stability and binding interactions through the lens of evolution: A dynamical perspective. Curr. Opin. Struct. Biol..

[61] Wang H., Lu J. (2020). A review on particle size effect in metal-catalyzed heterogeneous reactions. Chin. J. Chem..

[62] Guo H., Yu Y., Hong Z., Zhang Y., Xie Q., Chen H. (2021). Effect of collagen peptide-chelated zinc nanoparticles from pufferfish skin on zinc bioavailability in rats. J. Med. Food.

[63] Chen J., Li J., Li Z., Yi R., Shi S., Wu K., Li Y., Wu S. (2019). Physicochemical and functional properties of type I collagens in red stingray (*Dasyatis akajei*) skin. Mar. Drugs.

[64] Gbogouri G., Linder M., Fanni J., Parmentier M. (2004). Influence of hydrolysis degree on the functional properties of salmon byproducts hydrolysates. J. Food Sci..

[65] De La Fuente-Betancourt G., García-Carreño F., Navarrete Del Toro M., Córdova-Murueta J.H., Lugo-Sánchez M.E. (2009). Protein solubility and production of gels from jumbo squid. J. Food Biochem..

[66] Capanoglu E., Kamiloglu S., Demirci Cekic S., Sozgen Baskan K., Avan A.N., Uzunboy S., Apak R. (2020). Antioxidant activity and capacity measurement. Plant Antioxidants and Health.

[67] Zhang F., Yang A., Ma S., Shang H., Wang Z. (2019). Antioxidant activity of extracts from *Uncaria scandens*. IOP Conference Series: Earth and Environmental Science.

[68] Li P., Lu Y., Long G., Li S., Li K., Jiang B., Wu W. (2023). Structural Characterization of Acid DES-Modified Alkaline Lignin and Evaluation of Antioxidant Properties. Forests.

[69] Bozin B., Mimica-Dukic N., Samojlik I., Jovin E. (2007). Antimicrobial and antioxidant properties of rosemary and sage (*Rosmarinus officinalis* L. and *Salvia officinalis* L., *Lamiaceae*) essential oils. J. Agric. Food Chem..

[70] Liu Y., Wu Q., Zhang J., Yan W., Mao X. (2024). Food emulsions stabilized by proteins and emulsifiers: A review of the mechanistic explorations. Int. J. Biol. Macromol..

[71] Zhang M., Hou Y., Liao A., Chen X., Wang Z., Zhao P., Pan L., Huang J. (2024). Preparation of wheat protein peptides-calcium chelate by the ultrasound method: Structural characteristics and bioactivity. Food Biosci..

[72] Matsubara M., Muraki Y., Suzuki H., Hatano N., Muraki K. (2024). Critical amino acid residues regulating TRPA1 Zn^2+^ response: A comparative study across species. J. Biol. Chem..

